# Breaking Lock-ins to Enable a Green Pharmacy

**DOI:** 10.1021/acs.est.5c12437

**Published:** 2026-02-18

**Authors:** Anna Shalin, Miriam L. Diamond, Zhanyun Wang

**Affiliations:** † Empa—Swiss Federal Laboratories for Materials Science and Technology, 9014 St. Gallen, Switzerland; ‡ Department of Earth Sciences, 7938 University of Toronto, Toronto, Ontario M5S 3B1, Canada; § School of the Environment, University of Toronto, Toronto, Ontario M5S 3E8, Canada; ∥ 27219 National Centre of Competence in Research (NCCR) Catalysis, 8093 Zürich, Switzerland

**Keywords:** drug development, green chemistry, benign by design, chemical lock-in, safe and sustainable by design

## Abstract

Environmentally hazardous pharmaceuticals continue to be produced, used, and released to the environment, despite growing recognition of the need for greener drug design. This study applies a “lock-in” analytical framework to examine how various interacting social, economic, political, and technological factors prevent the effective implementation of concepts such as “benign by design” in drug development. Using a combination of qualitative and quantitative methods, including analysis of US and European clinical trial records, we identify two critical lock-ins: (1) misaligned timing of resources and expertise among key actors that prevents early integration of environmental safety and sustainability considerations in drug design, and (2) incentive structures that promote new drug development over the redesign of existing pharmaceuticals. Notably, this analysis identifies barriers to greening both future and existing drugs on the market. Building on Meadows’ framework for effective interventions in complex systems, we propose a strategic approach for breaking lock-ins and present specific recommendations for key stakeholder groups including policymakers, academia, small biotech, big pharma, and financial institutions. Addressing these lock-ins is essential to unlocking the full potential of green innovation in pharmaceutical development, while providing for patient access to essential medicines.

## Graphical abstract

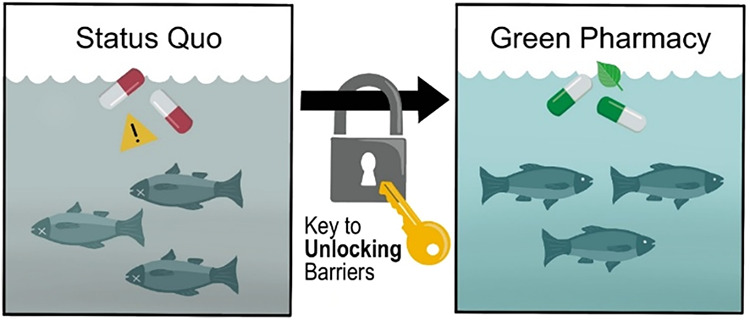

## Introduction

Access to pharmaceuticals has significantly improved over the past decades, leading to better patient outcomes and enhanced quality of life for many. However, the increasing production and consumption of drugs has also resulted in their widespread environmental contamination.^1–4^ This issue arises from factors across the entire life cycle of pharmaceuticals, including regular use, overprescription, inappropriate disposal, and limited availability of methods for and efficacy of treating human and animal waste, even with state-of-the-art treatment facilities (though recent advances in quaternary treatment technologies have improved the removal of micropollutants from wastewater, including many pharmaceuticals).^5,6^ Pharmaceuticals are designed to be biologically active at low concentrations. Toxic effects of pharmaceuticals and their transformation products in nontarget organisms are well-documented, ranging from molecular- to population-level impacts.^7–13^ Of great concern is the widespread presence of antimicrobial agents in the environment, which is increasingly thought to contribute to the escalation of antimicrobial resistance.^3,14^

Creative solutions are needed to mitigate environmental impacts while ensuring equitable patient access to lifesaving therapies.^5^ Scholars from various disciplines, including chemistry, environmental sciences, pharmaceutical sciences, social sciences, economics, and others have made important contributions to understanding aspects of this complex systems challenge (see SI Table S3 for a detailed literature overview).^5,15–29^ Early efforts to address pharmaceutical pollution include pharmaceutical take-back programs and end-of-pipe solutions such as advanced wastewater treatment technologies, currently possible in limited geographies.^5,6,30^ However, there is growing recognition that a more fundamental solution is needed—one that recognizes that the most effective solutions start at the design stage.^5,17,21,22^ The Twelve Principles of Green Chemistry, introduced by Anastas and Warner, provide a foundational framework for designing safer chemicals and synthesis processes, including pharmaceuticals, emphasizing the importance of chemical design that preserves efficacy of function while reducing toxicity and environmental persistence.^31^ This concept of benign/safe by design to develop nonhazardous pharmaceuticals through molecular design is increasingly recognized by key stakeholders, including academia, funding bodies, NGOs, governments, and industry.^5,17,18,29,32^ In practice, these concepts remain underutilized. Rather, many pharmaceutical developers focus on manufacturing sustainability metrics, such as reducing resource consumption through continuous manufacturing techniques and using green chemistry principles to replace hazardous chemicals in synthesis routes.^23,33,34^

Barriers and drivers to adopting environmental safety and sustainability considerations in pharmaceutical development have been explored in the literature (see SI Table S3). To date, much of this work has focused on specific stages of the pharmaceutical life cycle (e.g., synthesis), particular stakeholder groups (e.g., major pharmaceutical companies), or barriers and drivers specifically related to the development of new drugs.^5,15–29,35^ Comparatively less attention has been paid to addressing existing pharmaceuticals and to a holistic analysis of the entire complex system, including the roles and interests of different actors.

Our analysis, using a “lock-in” framework,^36^ takes a holistic approach to address these underexplored dimensions. This study aims to understand why environmental safety and sustainability has not yet been broadly incorporated into molecular design of pharmaceuticals, despite longstanding recognition of the importance of concepts such as benign-by-design and the Twelve Principles of Green Chemistry,^17,18,31^ and to identify where strategic interventions can most effectively break the systemic barriers to progress. The analysis reveals the interplay of current economic, social, technological and political factors that limit the integration of environmental safety and sustainability considerations at the molecular design stage. We summarize them into two key lock-ins. We contend that effective implementation and the true ″greening″ of the pharmacy requires addressing these lock-ins by recognizing and reshaping their underlying dynamics. Moreover, the current analysis distinguishes between barriers and drivers for existing versus new pharmaceuticals. To drive systemic change, we further propose a strategic approach that targets critical leverage points and actors through concerted action.^37^

## Lock-in 1: Misaligned Timing of Resources and Expertise in Integrating Environmental Safety and Sustainability in Pharmaceutical Development

The pharmaceutical development pipeline, typically spanning 10–15 years, begins with drug discovery and preclinical testing, where candidate compounds are identified and assessed. Three phases of clinical trials follow for promising candidates to evaluate safety, dosage, and efficacy in humans. Regulators review data from these trials as part of market approval. Once approved, any major changes that may substantially alter the identity, strength, quality, purity, or potency of a pharmaceutical must be resubmitted for regulatory review and approval before release to the market.^38,39^ Failure in drug development can occur at any stage of the process, preventing a drug candidate from advancing to the next stage. These failures may result from various reasons, including patient safety concerns, poor pharmacokinetics, lack of efficacy, lack of funds, or regulatory rejection.

The most effective point to integrate environmental safety and sustainability considerations (such as environmental screening toward “benign-by-design” chemical structures) is during the initial drug discovery and preclinical R&D stages, because this is the latest stage at which structural modifications to a compound can feasibly be made without necessitating major re-executions of earlier stages.

However, in practice, these considerations are often overlooked at this early phase. This stems from a misalignment in the timing and availability of expertise and necessary resources among the key players in pharmaceutical development—academia, biotech start-ups, and large pharmaceutical companies (hereafter “big pharma”)—who each operate at different points along the drug development pipeline and are driven by distinct goals and constraints. This misalignment is shaped by a mix of economic, social, technological, and political (including legislative) factors (Figure 1).

**Figure 1:**
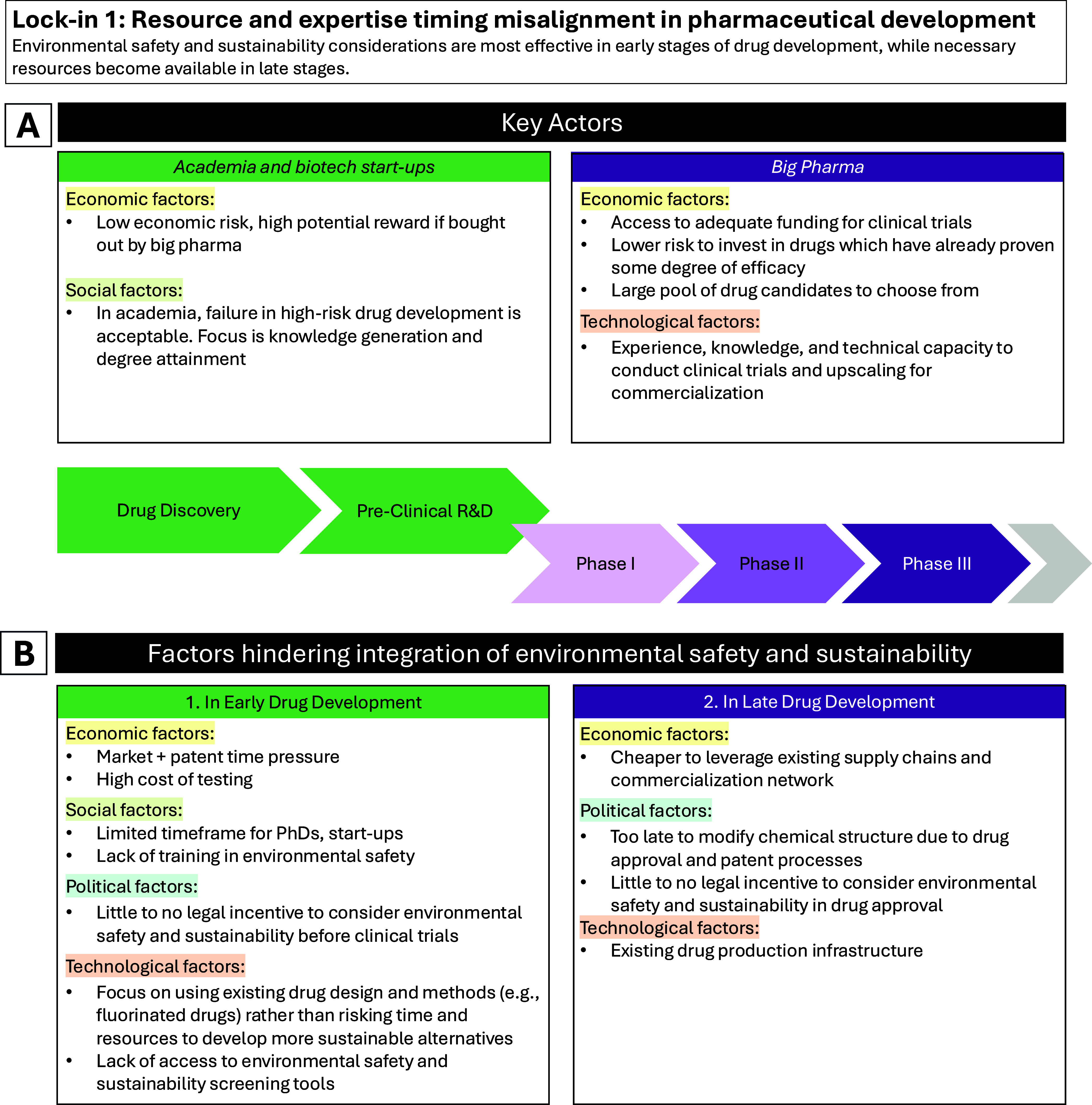
Lock-in 1: Resource and expertise timing misalignment in pharmaceutical development. Overview of the drug development process, key actors (panel A), and the economic, social, technological, and political (including legislative) lock-in factors leading to resource timing misalignment in integrating environmental safety and sustainability in pharmaceutical development (panel B).

Pharmaceutical development is marked by high attrition rates: only a small fraction of candidate drugs progress to preclinical testing out of thousands of compounds screened, and fewer yet reach the market.^40^ Different actors are involved at different stages of drug development. Using literature and clinical trials data (i.e., all records from the ClinicalTrials.gov database as of January 2024 for US data and February 2024 for European data, see SI Text S1 for methods), we found that academia and biotech start-ups dominate the early stages of pharmaceutical development (Box [Boxed-text box1], Panel A) due to multiple social and economic factors (Figure 1).
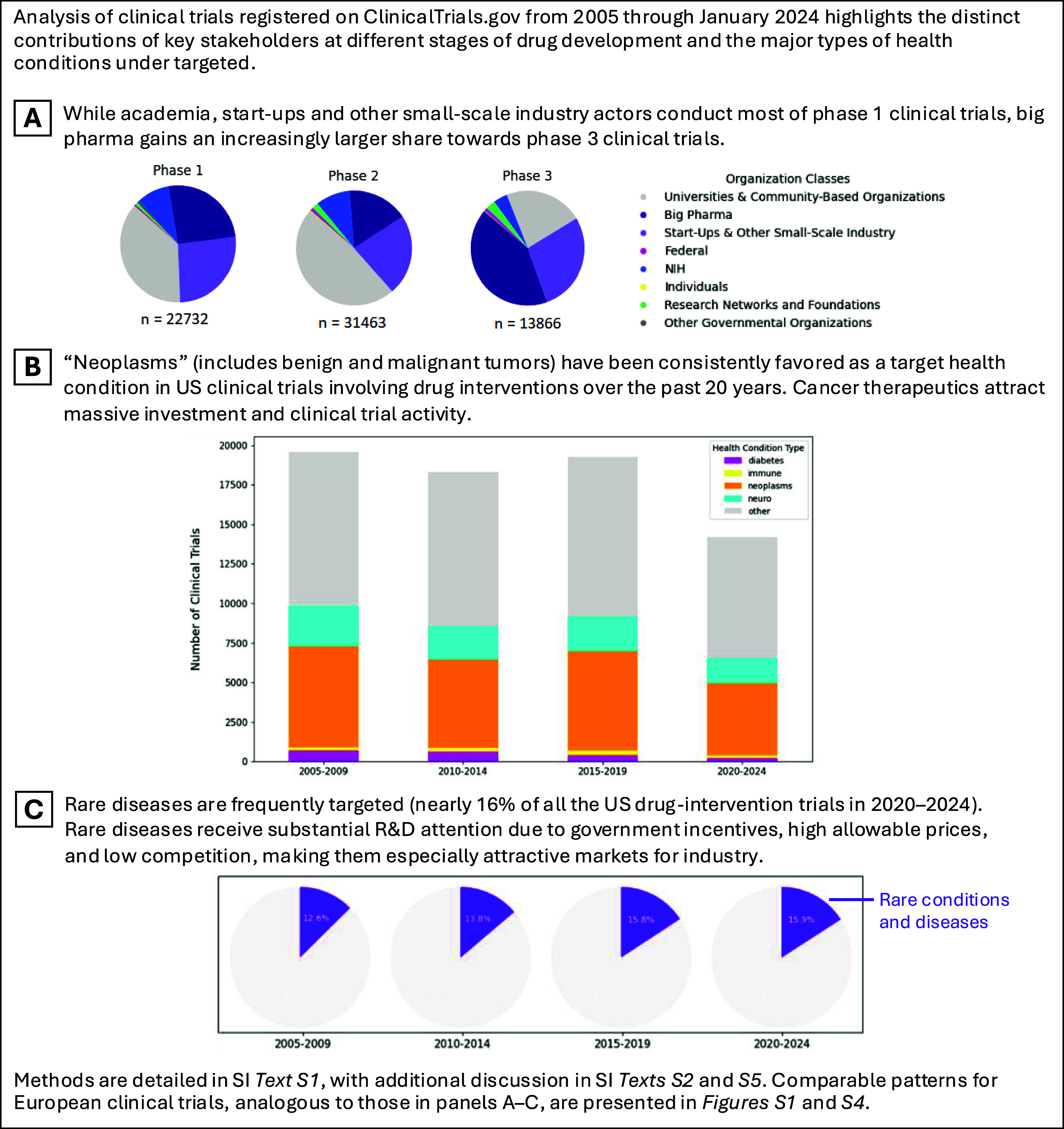

1 **Analysis of the US clinical trials data between January 2005 to January 2024 (see next page)**

Socially, failure in early drug development is acceptable in academia because the focus is on knowledge generation and degree attainment. Economically, both academia and start-ups benefit from diverse funding sources including public and institutional funds, which “de-risk” drug discovery while the potential for acquisition by big pharma provides a strong financial incentive for the research.^41,42^ In contrast, big pharma focuses on moving drugs to the market, which involves late-stage clinical trials and commercialization. They selectively acquire promising compounds from a broad pool of smaller entities who conduct high-risk research, thereby minimizing economic exposure and keeping shareholder interest (see SI Text S2 for additional supporting evidence for the roles of various actors in different stages of drug development).^43–46^

This dynamic creates a financial win-win for both early and late-stage actors. However, without strong regulatory (or other) incentives, this division of priorities across actors hinders the effective integration of environmental safety and sustainability considerations in pharmaceutical development at the appropriate time (Figure 1).

Regulations across jurisdictions require limited early testing in target organisms for clinical safety. However, these mandated tests do not address the broader environmental impacts of pharmaceuticals on diverse nontarget species, particularly on population- and ecosystem-level processes. Even where environmental risk assessments are mandated in drug approval processes, their influence remains limited. For example, in the EU, while environmental risk assessments are required when submitting a market application for a human drug, this assessment currently has no bearing on the approval of the pharmaceutical, and there are no penalties for noncompliance.^47^ Additionally, environmental safety and sustainability are not widely emphasized or taught in academic curricula outside of environmental sciences, leaving many researchers without the necessary awareness or background to achieve benign by design. Even if some researchers are aware, additional testing for environmental safety and sustainability is costly and has no immediate financial return, making it difficult to justify within tight funding cycles and fast publication-driven timelines. These factors collectively limit researchers in academia and biotech start-ups from fully integrating environmental safety and sustainability considerations into the early stages of drug development.

By the time big pharma acquires a drug candidate (typically after phase 2 clinical trials, see Box [Boxed-text box1], Panel A and SI Text S2), it is too late to make meaningful structural modifications to the drug. The clinical trial process does not allow for substantial chemical changes, as this would require new regulatory approval, adding years to development timelines and incurring significant costs. Given that patents typically last 20 years from filing (usually before clinical trials) and clinical trials can take a decade or more, companies are under pressure to maximize market exclusivity periods,^48–50^ focusing on rapid commercialization instead of addressing environmental concerns (see SI Text S3 for supporting evidence on patent expirations).

## Lock-in 2: Incentives Promote Development of Niche, New Pharmaceuticals over Redesign of Existing Drugs

Actors interested in drug development have three major options: improving existing drugs (including, but not limited to, sustainability improvements), novel drug discovery, and acquisition of drug discovery start-ups (SI Text S4). The manufacture of generic pharmaceuticals after patent expiry is a major component of the sector (see SI Text S3). However, because generics involve reproducing, rather than redesigning, existing pharmaceuticals, they offer only limited scope for changes to the molecular design. Hence, the role of generic manufacturing companies is not further discussed.

Redesigning existing pharmaceuticals, even those with known environmental hazards, is discouraged by high costs for redesigning and testing, and lengthy reapproval processes. Major alterations in chemical structure require costly new clinical trials with no guarantee of a return on investment, as the newly modified drug may not be approved. Also, redesign pertaining to environmental safety and sustainability may not be rewarded under existing patent systems. Companies can extend their market exclusivity periods through minor nonstructure-related modifications such as changes to formulation, dosage, or method of administration.^48,51^ However, such minor alterations likely cannot significantly improve environmental safety and sustainability. Additionally, major changes to the drug composition could disrupt existing supply chains and manufacturing processes. The market space for pharmaceuticals within established therapeutic areas also tends to be highly competitive, so potential financial gains are limited even with a newly redeveloped drug with better environmental performance.

In contrast, investing in novel drug discovery is more financially attractive, especially within niche therapeutic areas such as oncology and rare disease where governments have introduced incentives to drive innovation (see SI Text S5). Actors benefit from reduced competition, a plethora of supporting government subsidies, and guaranteed market exclusivity with new patent protection. Illustratively, of all US clinical trial submissions from 2020–2024, 32% were for oncology treatments and 16% for rare diseases (see Box [Boxed-text box1], Panels B and C). Actors with sufficient financial means (i.e., big pharma) can also acquire drug discovery start-ups that have already demonstrated some efficacy. This strategy offers a lower-risk, high-reward pathway, with the added advantage of rapid financial returns once the drug gains market approval.

In light of these preferable highly profitable options, the redesign of existing drugs (especially generics) is often dismissed as an option in drug development, and efforts to improve their environmental safety and sustainability are sidelined (Figure S2).

## Intertwined Lock-ins Require Holistic Solutions

Tackling lock-in 1 and 2 is essential toward “greening” the upstream aspects of drug development. The various contributions to understanding and solving aspects of this complex systems challenge proposed to date by scholars^5,15–29,35^ (SI Table S3) will be limited in their success if these core issues of lock-in are not addressed.

For novel pharmaceuticals, addressing lock-in 1 ensures that future drugs are designed to be inherently safe and sustainable by incorporating environmental considerations when it matters most—during early molecular design. For existing pharmaceuticals to be successfully revisited and improved in their environmental safety and sustainability, however, both lock-ins must be addressed. In this context, addressing lock-in 2 is key to ensuring that appropriate economic and regulatory incentives exist to justify and foster action on redesigning existing drugs, while addressing lock-in 1 ensures that adequate environmental considerations are incorporated into the redesign process.

## Strategies for Breaking Lock-ins Toward Transformative Change

To truly green the pharmacy and unlock the potential of designing safe and sustainable drugs to address pharmaceutical pollution in the environment, targeted actions are needed to overcome the two lock-ins. The goal for breaking lock-in 1 is to integrate and prioritize environmental safety and sustainability considerations in early drug development. For lock-in 2, the goal is to create incentives to encourage interest in improving existing drugs. To achieve these two goals, we have identified a list of actionable measures, inspired by Meadows’ framework on effective intervention points^37^ and the ideas of previous studies such as those of Kümmerer and Hempel on green and sustainable pharmacy^18^ (see SI Table S3 for a full list of related studies). The approach encompasses six types of actionable measures in order of increasing leverage: information flows, financial incentives, rules of the system, structure of the system, goals of the system, and mindset and paradigm. Because lower-leverage measures are more tangible and readily implementable by specific actors, most measures we have identified are listed in this category. However, these should be understood as foundations for moving toward the higher-leverage intervention points that represent deeper, systemic transformation. Supporting details for the strategic approach are provided in SI Text S6.

Figure 2 captures the strategic approach to breaking lock-ins with some measures listed for specific actors. Here, we summarize key takeaways by stakeholder group, noting that certain measures involve multiple stakeholders with distinct roles.

**Figure 2:**
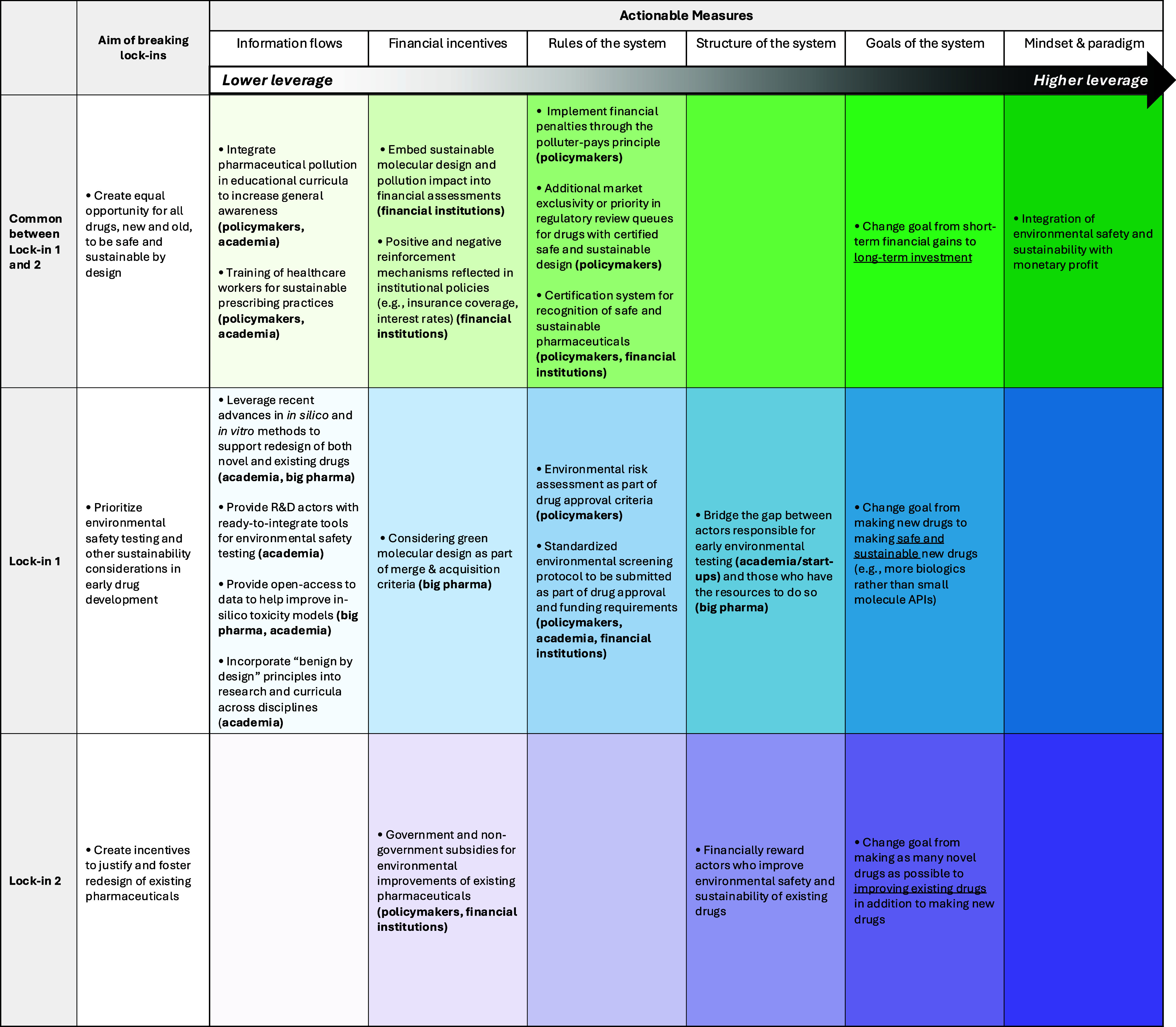
Strategies toward breaking lock-ins. Actionable measures for breaking lock-ins in the drug development pipeline are systemically explored using leverage points in complex systems, based on the conceptual framework by Meadows.^37^ Columns denote the progression of leverage points. Rows denote the lock-in being addressed. Actionable measures are listed accordingly in the matrix. Lower leverage measures (under “information flows”, “financial incentives”, and “rules of the system”) are more concrete and achievable by specific identified actors. They may be useful as steppingstones toward achieving higher leverage points (“structure of the system”, “goals of the system”, and “mindset and paradigm”), which describe more systemic change needed to truly “green” the pharmacy.

**Policymakers** have a crucial role in reshaping the rules of the system, with the authority to create and expand incentives that prioritize environmental safety and sustainability considerations in drug development. This could include mandating early stage environmental safety and sustainability testing (e.g., as a prerequisite to clinical trials)^15^ and/or ensuring that the outcome of the environmental risk assessment enters the decision leading to regulatory approval. One could argue that incorporating this in drug approval could further increase already high attrition rates. However, environmental considerations would represent one factor, rather than a singular gatekeeper, within a broader cost-benefit assessment. This would allow potentially life-saving therapies with unmet medical need to progress, while discouraging the approval of new drugs that offer limited therapeutic advantage yet pose comparatively greater environmental harm. A systematic evaluation of how inclusion of environmental risk assessment could influence approval outcomes, particularly relative to existing alternatives, would be a valuable direction for future research.

One practical way to operationalize this would be to make a standardized environmental screening protocol, developed in collaboration with environmental scientists, as an integral component of regulatory submissions and funding requirements. This protocol could incorporate recent advances in *in silico* and *in vitro* assessment methods (described in detail in the recommendations for researchers and academics).

Another potential regulatory approach is to implement financial penalties through the polluter-pays principle, as has been recently discussed in the revision of the EU Urban Waste Water Treatment Directive.^52–54^ In practice, this would require major contributors to micropollutants in urban wastewater, including the pharmaceutical sector, to bear part of the costs of necessary quaternary treatment, such as monitoring and installing advanced removal technologies.^52^ Although this regulatory intervention targets pollution at the “end of the pipe”, it can create an upstream incentive to design inherently safer drugs that would not require such treatment, or to redesign or substitute environmentally harmful existing pharmaceuticals.

There may be merit in examining the success of incentive structures for rare disease drug development as a model for encouraging innovation in environmentally safe and sustainable pharmaceuticals using positive reinforcement mechanisms (for more details on such incentive structures, see SI Text S5). This is especially important for the redesign of existing drugs, where major structural changes of the molecules could trigger new clinical trials and regulatory approval, creating significant economic barriers (lock-in 2) that need to be offset by incentives. Policymakers could introduce positive incentives such as extended market exclusivity periods or priority placement in regulatory review queues for certified sustainable pharmaceuticals.^16,21–23,25,27^ Developing a certification system for recognizing environmentally safe and sustainable pharmaceuticals is essential for this last point,^21,32^ which could also be leveraged by other actors (e.g., financial institutions who want to prioritize investing in green pharma). In addition, government subsidies could be granted for greening existing pharmaceuticals, to move beyond the existing financial subsidies for developing drugs in niche (incentivized) areas such as oncology and rare disease (see SI Text S5).

In sum, regulatory reforms offer important opportunities to better align pharmaceutical governance with environmental priorities. Importantly, policy coherence analysis is a useful tool to ensure that changes to regulations do not inadvertently reinforce existing lock-ins or create new ones.^55–57^

Lastly, with respect to increasing information flows, policymakers can promote inclusion of pharmaceutical pollution and solutions as part of educational curricula, including the training of healthcare workers to reduce unneeded pharmaceutical prescriptions, and to encourage preferential prescription of environmentally safer drugs, where appropriate.^5,24,25,27,28,32^

**Researchers and academics** can play a pivotal role in broadening information flows across disciplines and enabling practical implementation of approaches to integrate environmental safety and sustainability into the molecular and process design. Recent advances in *in vitro* and *in silico* tools, summarized by Castiello et al., now support early identification of potentially hazardous pharmaceuticals.^35^ These include high-throughput screening platforms (e.g., ToxCast, Tox 21), computational toxicity prediction models, databases containing biodegradation data, and predictive tools for estimating environmental persistence.^35,58–62^ Such tools have enabled redesign of known persistent pharmaceuticals (e.g., greener analogues of EDTA, some β-blockers, and ciprofloxacin),^63–67^ illustrating that safer molecular alternatives are achievable with modest alterations.^35^ While these approaches are continuously evolving to broaden their scope and applicability, they represent a substantial resource that warrants further investigation for safe and sustainable molecular and process design, especially for revisiting existing drugs. Environmental specialists could help adapt such tools so that actors in drug development with limited environmental expertise can readily integrate and utilize them in existing workflows.^22,32^

Academics are also encouraged to incorporate “benign by design” thinking, among others, in research and as part of early university curricula promote their broader uptake and routine integration, not only in environmental fields, but across disciplines. In addition, they can help accelerate progress by openly sharing data relating to environmental safety and sustainability screening following FAIR data sharing principles, and helping regulatory agencies in developing clear and relevant environmental safety and sustainability criteria for pharmaceuticals.^22,24,25,27,29^

**Big pharma**, with their substantial technological and financial resources, are also well-positioned to support early stage environmental safety and sustainability testing and green drug design. This support can take the form of direct collaborations with academia and start-ups, or indirect contributions such as sharing open-access data and tools with the broader scientific community.^22^ The latter approach could significantly accelerate the development of more robust in-silico models for predicting environmental safety and sustainability, ultimately enabling greener innovation across the drug development pipeline.^21,22,26^

Big pharma also has an opportunity to champion green molecular design by incorporating environmental safety and sustainability criteria in their merger and acquisition portfolios. With supportive regulatory frameworks and well-aligned financial incentives from policymakers and financial institutions, this approach could not only drive green innovation but also position companies as leaders in novel drug development. For example, reframing environmental safety and sustainability as an investment could better position pharmaceutical companies for long-term financial gains.

**Financial institutions** also play a key role in shaping industry behavior through investment strategies. The integration of environmental safety and sustainability criteria into financial portfolios has already been shown to influence corporate decision-making, as seen with environmental, social, and governance (ESG) performance metrics.^68^ A similar opportunity exists to incorporate pollution mitigation into financial assessments. This has been demonstrated recently by major insurance companies denying coverage of business claims linked to PFAS.^69^ For pharmaceuticals, companies that continue to manufacture highly environmentally hazardous molecules could be disincentivized with higher insurance rates, or denial of coverage, underpinned by strong evidence demonstrating harm to ecosystem health^2–4^ (this could be particularly useful for addressing lock-in 2). Financial institutions can offer positive reinforcement for sustainable business behavior. For example, financial incentives such as grants, favorable interest rates on loans, or tax benefits could be given to start-ups aiming to improve environmental safety and sustainability of existing drugs.^25,27^

Overall, the greening of the pharmacy is at a critical juncture: promising solutions have been proposed, yet systemic barriers continue to limit their effectiveness. Addressing lock-ins using targeted leverage points is necessary to unlock the full potential of these innovative ideas, ensuring patient access to essential drugs while fostering a pharmaceutical sector that is both forward-looking and environmentally sustainable.

## Supplementary Material





## Notes

Information about methods to collect and analyze clinical trials data, roles of actors at different stages of drug development, patent expirations, barriers and drivers for different options in drug development, rare disease and oncology as key focuses of new drug discovery, Meadows’ (1999) leverage points for complex systems related to drug development, list of big pharma companies and their subsidiaries, and literature review of related studies (PDF)
List of big pharma (Table S2) and an overview of literature review (Table S3) (XLSX)
