# Supplementary material for: Breaking Lock-ins to Enable a Green Pharmacy
Source: Environ Sci Technol. 2026 Feb 18;60(8):5960–9. doi: 10.1021/acs.est.5c12437 (PMC12961740; doi:10.1021/acs.est.5c12437)
Supplement: Supplementary file 1 [file es5c12437_si_001.pdf]

Supporting Information

Breaking Lock-ins to Enable a Green Pharmacy

Anna Shalin[1,2], Miriam L. Diamond[2,3], Zhanyun Wang[1,4]*

[1]Empa—Swiss Federal Laboratories for Materials Science and Technology, 9014 St. Gallen, Switzerland.

[2]Department of Earth Sciences, University of Toronto, Toronto, Ontario M5S 3B1, Canada.

[3]School of the Environment, University of Toronto, Toronto, Ontario M5S 3E8, Canada.

[4]National Centre of Competence in Research (NCCR) Catalysis, 8093 Zürich, Switzerland

*Corresponding author. Email: zhanyun.wang@empa.ch

Summary: 29 pages, 6 figures, 3 tables (see Tables S2 and S3 in Excel spreadsheet)

Contents

List of texts

Text S1. Methods to collect and analyze clinical trials data ········ S3
Text S2. Supporting evidence for roles of actors in different stages of drug development ········ S8
Text S3. Supporting evidence on patent expirations········ S11
Text S4. Supporting evidence barriers and drivers for options in drug development ········ S13
Text S5. Supporting evidence on rare disease and oncology as key focuses in new drug discovery ········ S14
Text S6. Supporting information on Meadows' (1999) leverage points for complex systems · S22

List of tables

Table S1. Description selected health condition ········ S5
Table S2. List of companies included as big pharma, and their subsidiaries ········ Excel S2
Table S3. Literature review of related studies ········ Excel S3

List of figures

Figure S1. Distribution of actors in clinical trial submissions ········ S8
Figure S2. Options for drug development with relevant barriers and drivers ········ S11
Figure S3. Overview of health conditions targeted in US clinical trials ········ S16
Figure S4. Overview of health conditions targeted in European clinical trials ········ S17
Figure S5. Overview of health conditions targeted in US clinical trials by actor ········ S18
Figure S6. Detailed overview of health conditions targeted in clinical trials ········ S19
SI References ········ S25

Text S1. Methods to collect and analyze clinical trials data

Extraction of US clinical trials data. The ClinicalTrials.gov database, maintained by the US National Institutes of Health, is a collection of clinical research studies with information about their study designs and results. The purpose of using the database was to collect publicly available information on the key actors involved in clinical research studies (e.g., academia, industry, etc.) and their role in different stages of testing (e.g., phase 1, 2, 3 clinical trials), as well as information on targeted health conditions / therapeutic areas (e.g., neoplasms, diabetes, etc.).
The database contains information on studies primarily based in the US but also includes some studies from over 200 countries. Sponsors and investigators from these other countries can voluntarily list studies in the database. In the US, several laws require sponsors and investigators to submit information. For instance, Section 801 of the Food and Drug Administration Amendments Act (FDAAA 801) requires responsible parties to register clinical trials and publish results to ClinicalTrials.gov, applying to trials of drug, biological, and device products since 2007.[1] On a global level, the WMA Declaration of Helsinki, WHO International Clinical Trials Registry Platform, and EU Clinical Trials Directive are other examples of key laws and policies requiring clinical trial registration.[1]

While the database does not contain information on every clinical trial ever conducted globally, it is commonly regarded as the most comprehensive collection of clinical trials available in the public domain, which is why it was selected as the main reference for clinical trials data in the current study.

Data for all studies with the search term "Location" = "United States" were downloaded on January 22, 2024. For the European analysis, data for all studies from the following countries were collected on February 8, 2024: Albania, Austria, Belgium, Bulgaria, Bosnia, Croatia, Czech Republic, Denmark, Estonia, Finland, France, Germany, Greece, Hungary, Iceland, Ireland, Italy, Latvia, Lithuania, Luxembourg, Macedonia, Montenegro, Netherlands, Norway, Poland, Portugal, Romania, Serbia, Slovakia, Slovenia, Spain, Sweden, Switzerland, and the UK.

Selection of drug intervention studies. All studies with at least one drug intervention were included in the analysis (77,916 studies for the US and 124,742 studies for European countries). Examples of other intervention types include medical devices, procedures, vaccines, and

noninvasive approaches such as diet, which were excluded. A single study could have several different types of intervention, and the drug intervention was not necessarily always the main effect under investigation. For example, a clinical trial could be testing the effectiveness of a new diet in comparison to the conventional/established drug treatment. In such a case, the clinical trial is not representative of a new drug investigation. Due to the way the data were recorded, it was not possible to systematically exclude such "false-positive" studies from our analysis, nor was it feasible to manually remove them due to the size of the dataset. For this reason, the first 500 studies were manually checked, and only 3.8% were considered false positives under these conditions, which was deemed an acceptable degree of uncertainty.

Further classification of industry-led studies. The identities of the party responsible for each clinical trial were readily available in the database. However, for entries labelled as "industry" further differentiation was needed for the purpose of the current study. The goal was to differentiate between clinical trials submitted by major pharmaceutical companies and smaller companies including biotech start-ups. To address this, further classification of industry-led studies was conducted.

A list of "big pharma" companies, here defined as organizations with annual revenues above $1 billion, was created. To compile the initial list, a roster of publicly traded pharmaceutical and biotech companies generating annual revenues of $1 billion or more was assembled, amounting to 109 companies as of January 2024.[2] Manual revision during data cleaning led to the identification of an additional 25 companies with overall annual revenues above $1 billion. Additionally, synonyms and acronyms for each company were collected and included manually. Clinical trials led by any of the companies from this list were considered to be "big pharma" (SI Table S2).

Addressing subsidiaries. Given the common practice of large pharmaceutical companies acquiring smaller companies and start-ups, it was critical to discern whether the clinical trial was conducted before or after the company acquisition. If a company is acquired before clinical trials, they could potentially gain access to the resources and funding needed for environmental safety and sustainability testing and considerations provided by big pharma.

A manual desk search for each of the 134 identified big pharma companies was conducted to identify potential subsidiaries and their respective acquisition dates. If the acquisition date was before the submission date of the clinical trial, the study was labelled as "big pharma". If the

acquisition date was after the submission date of the clinical trial, the study was labelled as a start-up/small-scale industry.

Standardization and grouping of health conditions. While the health condition(s) targeted in each clinical trial were readily available in the database, categorization presented challenges due to inconsistencies in reporting. For instance, terms such as “diabetes type 2” and “type 2 diabetes” were treated as separate conditions despite being synonymous. To resolve this issue, the International Classification of Diseases 11th Revision (ICD-11) database was used to classify health conditions. Using the ICD-11 API coding tool, each health condition was inputted into the search tool individually, and the best matching ICD-11 standardized name and identification code were recorded. This allowed for the consistent naming of identical health conditions.
To gain a broader perspective of disease types, the ICD-11 tabulation spreadsheet was consulted, which describes the hierarchy of disease classification. This allowed for the grouping of specific health conditions under broader disease categories. For instance, terms such as “Leukaemia, unspecified” and “Squamous cell carcinoma of bronchus or lung” were collectively classified as “Neoplasms”.

During manual review, it became apparent that certain cancer types (and only this category of disease) were coded as “extension codes” rather than being categorized as “neoplasms” under Chapter 02 of the ICD classification scheme. To address this discrepancy and to accurately classify these data points, additional search criteria were checked ["cancer", "neoplasm", "malignant", "benign", "neoplasms", "leukaemia", "leukemia", "tumor", "tumour", "malignancy", "cancerous", "lymphoma", "oncology"] and relevant extension codes were re-coded to the appropriate “neoplasm” chapter.

The differentiation of common vs. rare diseases was also relevant for the study. The US Orphan Drug Act defines orphan drugs as a drug for a rare disease or condition that affects fewer than 200,000 people nationwide.[3] Examples include cystic fibrosis, Tourette’s syndrome, and amyotrophic lateral sclerosis (ALS).

Information on whether studies targeted a rare disease was not readily available in the ClinicalTrials.gov database. However, ICD-11 codes collected earlier could be used to identify

rare diseases using cross-referencing mapping files available from Orphanet, an online platform dedicated to providing access to key information pertaining to orphan diseases and orphan drugs.

Text S2. Supporting evidence for roles of actors in different stages of drug development

Evidence based on clinical trials data. Data availability in the early stages of drug development is scarce due to the maintenance of confidentiality for market competitiveness and lack of legal obligation to disclose this information. In contrast, data on human clinical trials are readily available. Submissions of clinical trials data offer a glimpse into the role of different actors at different stages of drug development.

Figure S1 summarizes the changes in the distribution of actors responsible for different phases of clinical trials. The main finding is that while academia, start-ups and other small-scale industry actors conduct the majority of phase 1 clinical trials, big pharma gains an increasingly larger share towards phase 3 clinical trials.

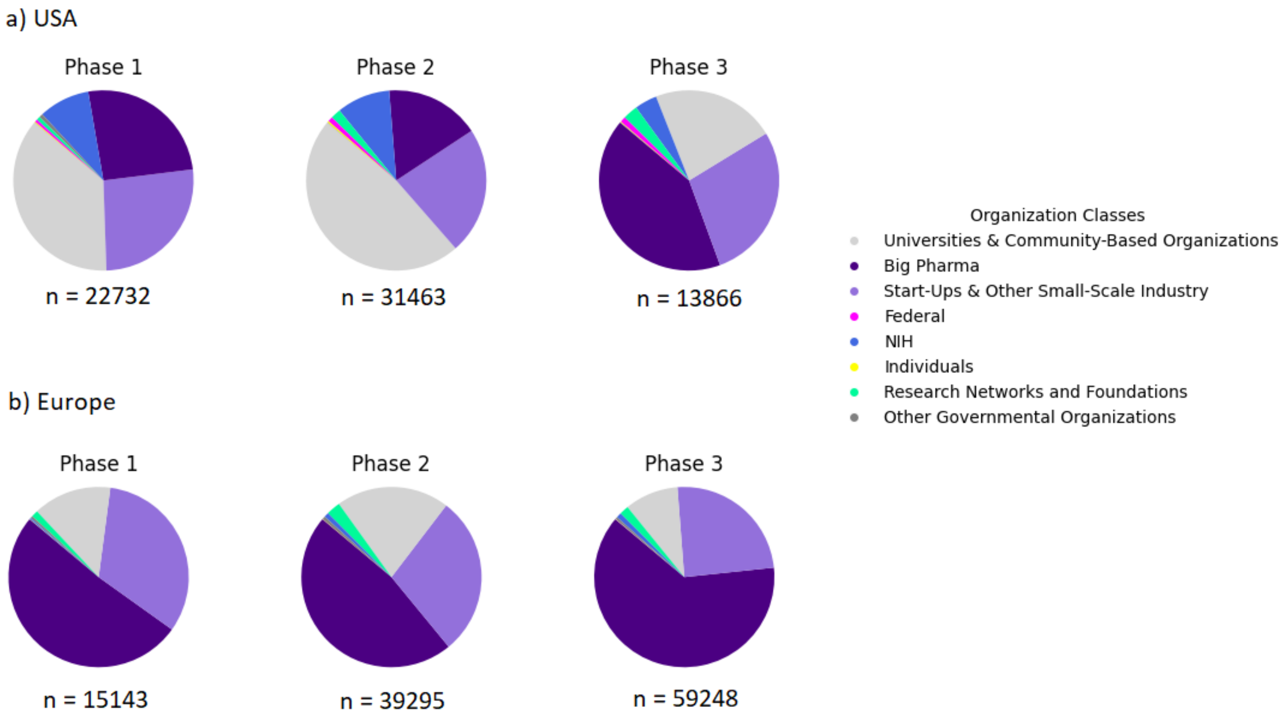

Figure S1. Distribution of actors responsible for all drug intervention clinical trial submissions to the ClinicalTrials.gov database, by clinical trial phase (as of Jan. 2024 for US data and Feb. 2024

for European data). Top row of pie charts represent US clinical trials; bottom row represents European clinical trials.

For European clinical trials, a similar trend occurs where big pharma's share of trials from phase 1 to phase 3 grows substantially (Fig. S1). However, a key difference relative to the US dataset is that European big pharma has a much larger share of studies conducted overall across all phases. A possible explanation for this discrepancy is that drugs originating from US start-ups or academia could be bought out in the US, and then subsequently brought to Europe after acquisition. Another possible reason for this difference between European and US data is that the recording of European data in the US ClinicalTrials.gov database could be biased. While all clinical trials in the US, regardless of the responsible party, are required to be reported in the database, European researchers do not have this obligation. Indeed, smaller research institutes and start-ups may not be aware of the database or may not see a reason to voluntarily report their findings. Regardless, the US and European datasets show similar trends of increasing share of big pharma towards the later stages of clinical trials.

Evidence based on literature. The proposed structure and role of actors in the drug development process dates back several decades in the literature; DiMasi's (2000) study on new chemical entity (NCE) approvals in the US from 1963 to 1999 noted a decrease in self-originated NCE approvals (in other words, drugs which have been developed by one firm), indicative of an increasing number of compound acquisitions.[4] For instance, all of the antineoplastic drug approvals submitted by Bristol-Myers Squibb were acquired drugs.

Recent studies show similar trends in big pharma acquisitions; in their study investigating pharmaceutical innovation activities from 2016 to 2019, Jung et al. (2020) highlighted that the top 20 companies possessed at least 25% of the drugs in each stage above phase 1, which then increased to up to 70% of drugs on the market.[5]

Large pharmaceutical companies have ample financial resources to support buyouts of smaller biotech start-ups. In 2022, the 18 major biopharma companies were estimated to have \$1.72 trillion in merger and acquisition (M&A) capacity.[6] Dealmaking capacity for the industry was estimated to be \$1.37 trillion in 2024.[7] While these numbers are indicative of significant potential financial

capacity, there is also a clear trend that substantial investments are being made in M&A, and that resources allocated to these activities are increasing annually. For instance, pharma and life sciences M&A investment rose from $142 billion in 2022 to $191 billion in 2023.[7]

Pharmaceutical companies need to introduce new drugs every year to sustain average industry growth.[8] Wajid et al. (2022) explained that merger and acquisition are a result of the failure to innovate.[8] Indeed, firms practicing merger and acquisition show more favorable corporate productivity when compared to companies that do not.[9]

Ultimately, the clinical trials data and evidence found in the literature point to the same trend: big pharmaceutical companies are buying out smaller biotech start-ups in the late stages of drug development. This suggests that academia and start-ups are the key players in early drug development, and big pharma becomes increasingly involved in the process towards later stages of clinical trials.

Text S3. Supporting evidence on patent expirations

Actors in drug development face time pressures arising from looming patent expirations. Once a drug patent is granted, the patent holder has exclusive rights to manufacture, use, and sell their product for a limited period, typically 20 years from the patent filing date. Once this exclusivity period ends, other companies can seek approval from regulatory agencies to produce and sell generic versions of the drug. The entry of generics into the market results in a drastic reduction in profitability for pharmaceutical companies. Since patent granting occurs well before drug marketing, the lengthy development and approval process usually leaves only 6–10 years of effective patent protection on the market.[10] Therefore, even early-stage drug developers face significant time pressure.

When the patent of a brand-name drug expires, competitors gain the right to sell the same active pharmaceutical ingredient as a generic drug. Generic manufacturers need to only provide evidence that their drug is medically equivalent to their brand-name counterpart and forego the lengthy and expensive processes of clinical trials and drug approvals.[11]

However, penetrating the market for generics is difficult in practice due to efforts by the brand-name drug manufacturers to keep competitors off the market. The entry of generics into the market typically results in substantially lower prices for both the brand name and generic drug. When generics first enter the market, prices can drop as much as 20%, and with the entry of multiple generics, this drop can increase to 85%.[12,13] Pharmaceutical companies thus have a clear financial incentive to delay the entry of competitors by as much as possible. Even a few months of extended market exclusivity can be worth hundreds of millions of dollars, especially for blockbuster drugs which can reach annual revenues in the billions.[12] Maximizing the market exclusivity period is synonymous with maximizing profitability. Average revenues decline by over 80% within the first two years of losing market exclusivity.[14]

A resulting pattern that emerges in the current drug ecosystem is the "evergreening" of drugs, where patent-holders approaching their expiration date create barriers to ward off the entry of generic competitors. The creation of patent thickets is one commonly used strategy. A patent thicket is an overlapping set of intellectual property rights requiring interested competitors to reach licensing deals for multiple patents.[15] While an interested competitor may have the right to produce

a generic drug, the costs associated with cutting through the patent thicket would outweigh any potential monetary gains to be made from generic production.[15,16]

Another approach to evergreening is to make minor modifications to the drug, which in turn warrant new patent applications or patent extensions.[12,17] Such modifications could include changes to formulation, dosage, or method of administration.[12,17] As a result, companies can receive additional years of market exclusivity.[12,17] Indeed, many of the patent applications reflect evergreening behaviour: Feldman (2018) found that 78% of drugs associated with new FDA patents were not novel drugs, but existing drugs.[12] Extending market exclusivity through patents was found to be particularly critical for blockbuster drugs: of the top 100 best-selling drugs, over 70% have had their patent protection extended at least once, and nearly 50% have had multiple extensions.[12]

With respect to creating generics for drugs where other generic manufacturers already have managed to penetrate the market, there is little financial incentive to do so due to existing competition and increasingly smaller profit margins (i.e., profit margins for generics manufacturers worldwide have decreased from 19.9% in 2016 to 12.8% in 2019).[18]

Thus, patent thickets, small profit margins, and existing competition are barriers that disincentivize new actors from choosing generics as a route for drug development.

Text S4. Supporting evidence barriers and drivers for different options in drug development

Academia and start-ups are incentivized to invest in new drug development, whereas big pharma benefits the most from acquisition of new drug discovery start-ups. Improving existing drugs offers fewer financial incentives, leading to unintentional sidelining of environmental improvements. Relevant barriers and drivers are summarized in Figure S2.

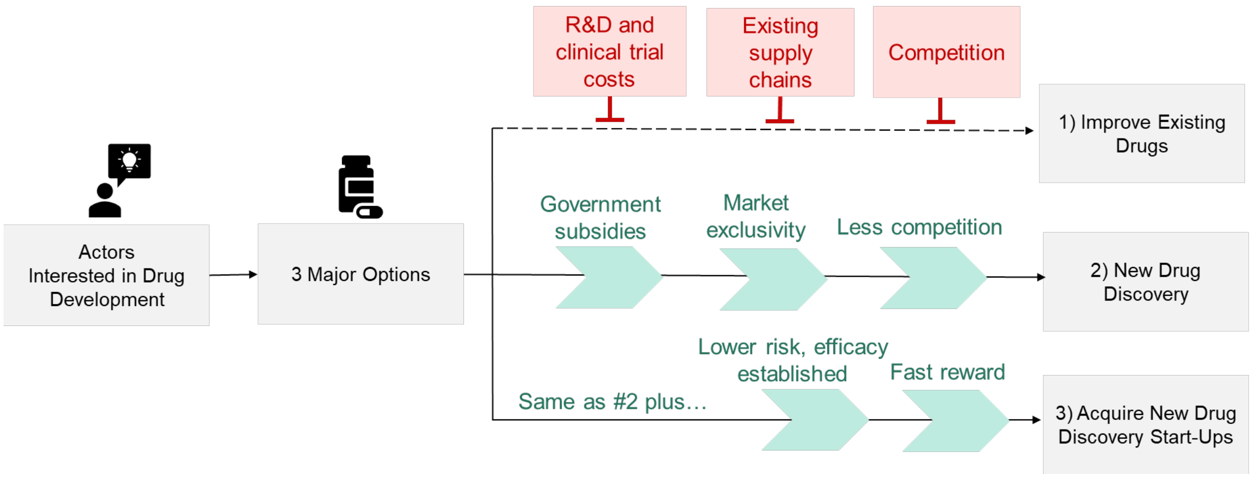

Figure S2. Possible options for actors interested in drug development with relevant barriers and drivers.

Text S5. Supporting evidence on rare disease and oncology as key focuses in new drug discovery

Neoplasms & Oncology. From the perspective of high sociodemographic index (SDI) countries, there is a public health need for the development of cancer therapeutics based on high disability-adjusted life years (DALYs). It is estimated that by 2026, approximately $307 billion USD will have been spent on cancer drug R&D globally, with a strong focus (55%) on treatments for cancers of the breast, lung, prostate, and multiple myeloma.[19] Figures S3 and S4 highlight the large focus on clinical trials with drug interventions targeting neoplasms, especially among university research, the National Institutes of Health (NIH), and other research networks and foundations (Fig. S5).

However, Sullivan (2023) argues that the current system focuses disproportionately on maximizing production and profitability rather than prioritizing clinical and societal benefits.[19] For instance, only 35% of the solid cancer therapies approved by the FDA since 2017 were graded as delivering clinically meaningful benefit.[19] Cancer research has become largely focused on biopharmaceutical R&D and discovery science, with a lack of real-world evidence studies to validate the effectiveness of emerging medicines.[19]

Oncology is now widely recognized as a "growth market for investors".[20] Many of the largest biopharma merger and acquisition deals reflect this; for example, Pfizer bought out oncology-focused pharmaceutical manufacturer Seagen in 2023 for $43 billion USD.[21] Pfizer estimated that Seagen would generate approximately $10 billion USD in 2030, continuing to drive profits by expanding its existing oncology portfolio.[21] In 2018, oncology had the highest global revenue amongst therapeutic drugs, generating $99.5 billion USD.[22] Highly prevalent diseases in high SDI countries such as cancer have thus shown to be financially desirable targets for drug development.

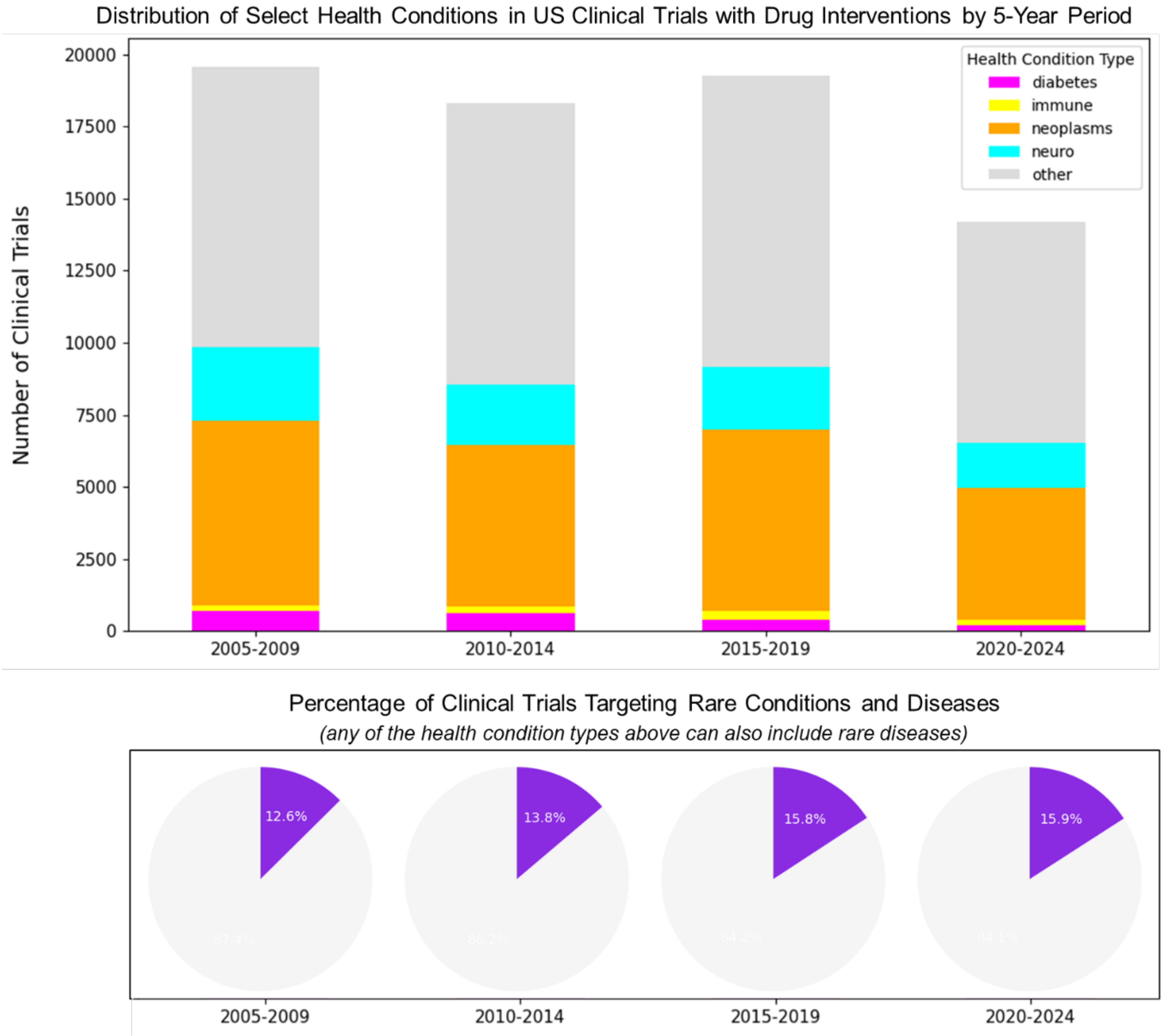

Figure S3. Overview of types of health conditions targeted in US clinical trials with drug interventions. Top graph: number of clinical trials in the US for selected health conditions (i.e., diabetes, immune, neuro, neoplasms, other) by 5-year period. Bottom graph: percentage of trials targeting rare health conditions and diseases by 5-year period. Accompanying Fig. S6 provides a detailed breakdown of the "other" category of health condition types in this figure. Descriptions of the selected health condition types can be found in Table S1.

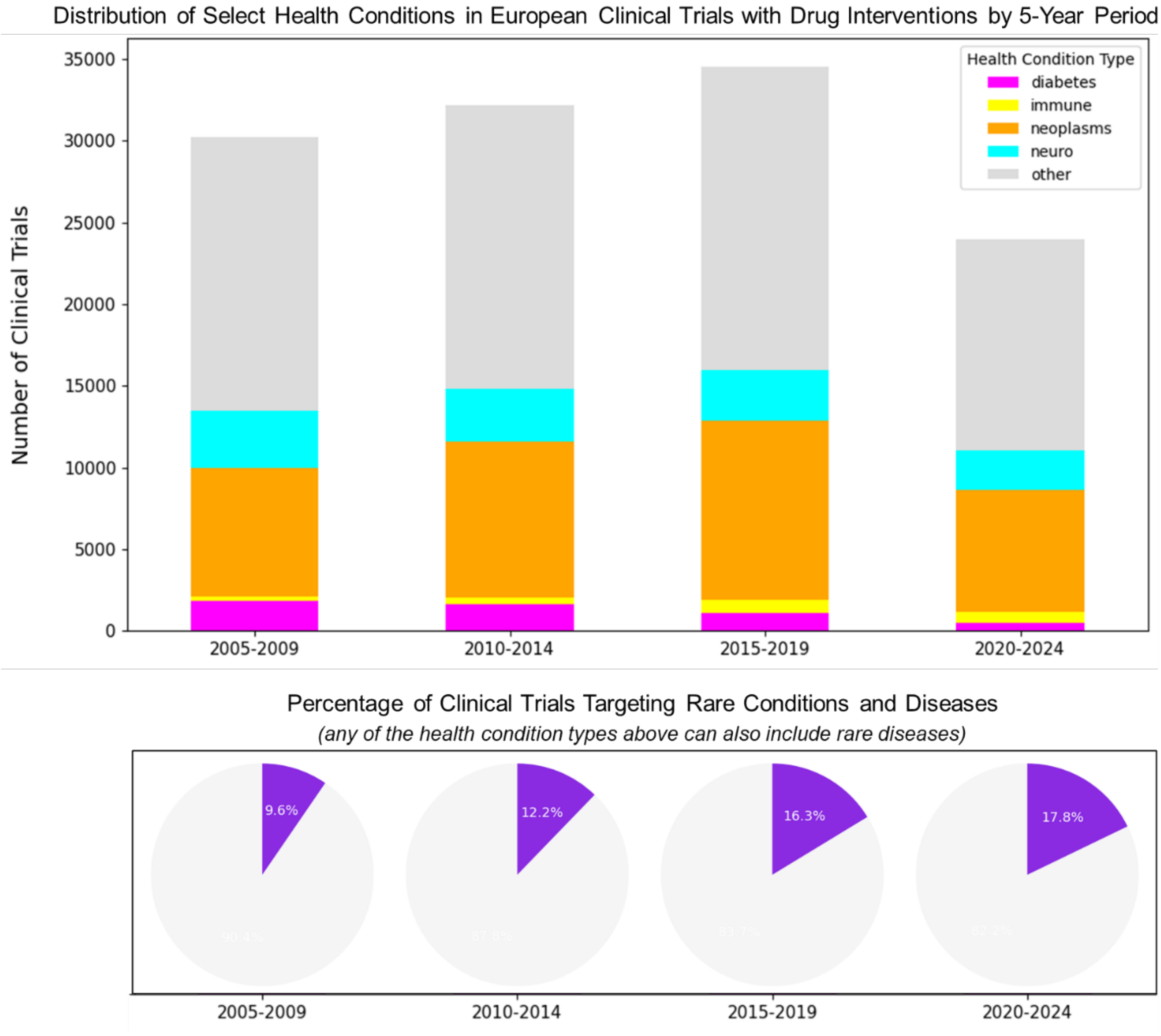

Figure S4. Overview of types of health conditions targeted in European clinical trials with drug interventions. Top graph: number of clinical trials in Europe for selected health conditions (i.e., diabetes, immune, neuro, neoplasms, other) by 5-year period. Bottom graph: percentage of trials targeting rare health conditions and diseases by 5-year period. Accompanying Fig. S6 provides a detailed breakdown of the "other" category of health condition types in this figure. Descriptions of the selected health condition types can be found in Table S1.

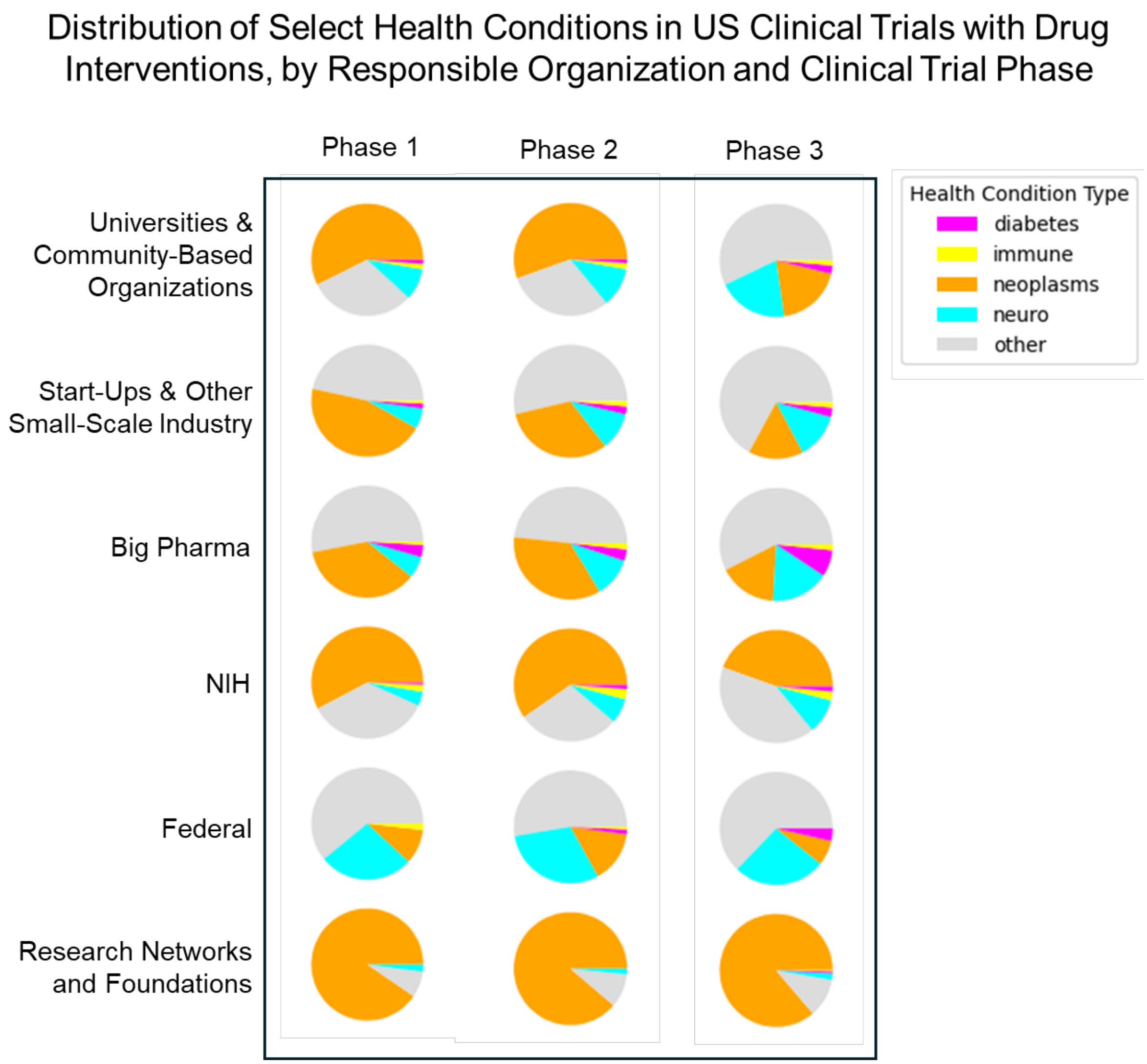

Figure S5. Analysis of the health conditions targeted in US clinical trials with drug interventions shows a large focus on neoplasms, especially for universities, the NIH, as well as research networks and foundations.

Table S1. Description of specific health conditions included in each of the selected health condition types in Figures S3, S4, S5.

**Health Condition Type**	**What is included**
Diabetes	Type 1 diabetes mellitus (ICD-11 code 5A10)<br>Type 2 diabetes mellitus (ICD-11 code 5A11)<br>Malnutrition-related diabetes mellitus (ICD-11 code 5A12)<br>Diabetes mellitus, other specified type (ICD-11 code 5A13)<br>Diabetes mellitus, type unspecified (ICD-11 code 5A14)
Immune	All ICD-11 codes classified under “Diseases of the immune system”
Neuro	All ICD-11 codes classified under “Diseases of the nervous system” and “Mental, behavioural or neurodevelopmental disorders”
Neoplasms	All ICD-11 codes classified under “Neoplasms” and all ICD-11 codes starting with “XH” under “Extension Codes”, plus any missed health conditions which have any of the following key words: “cancer”, “neoplasm”, “malignant”, “benign”, “neoplasms”, “leukaemia”, “leukemia”, “tumor”, “tumour”, “malignancy”, “cancerous”, “lymphoma”, “oncology”

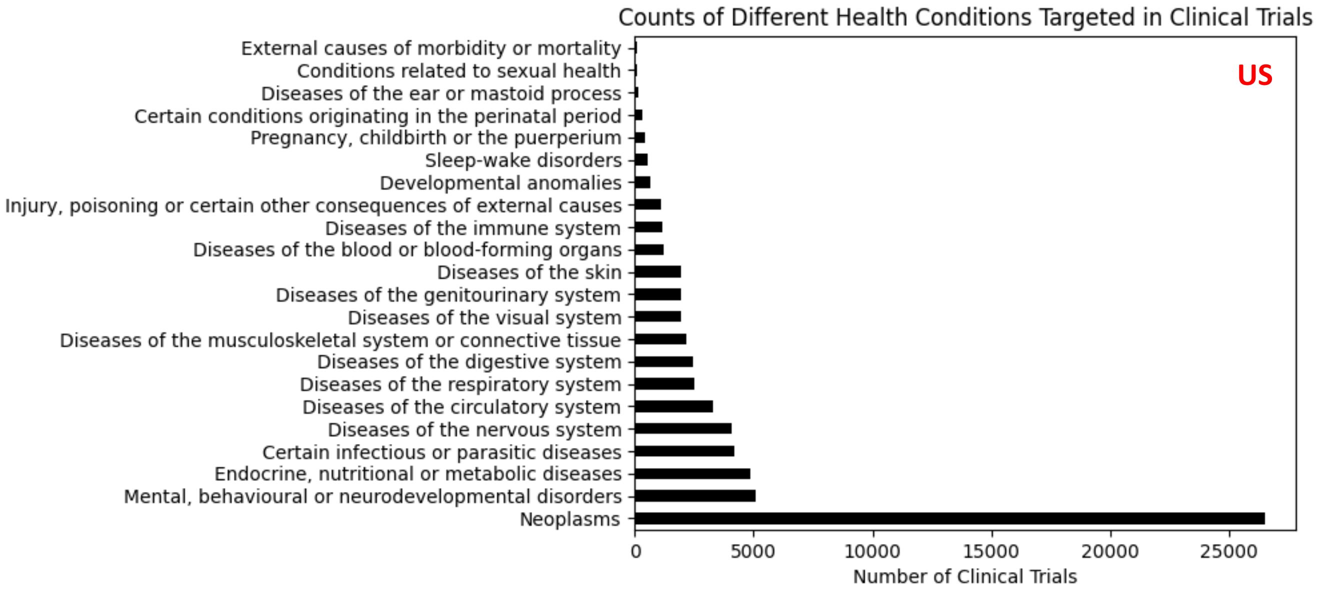

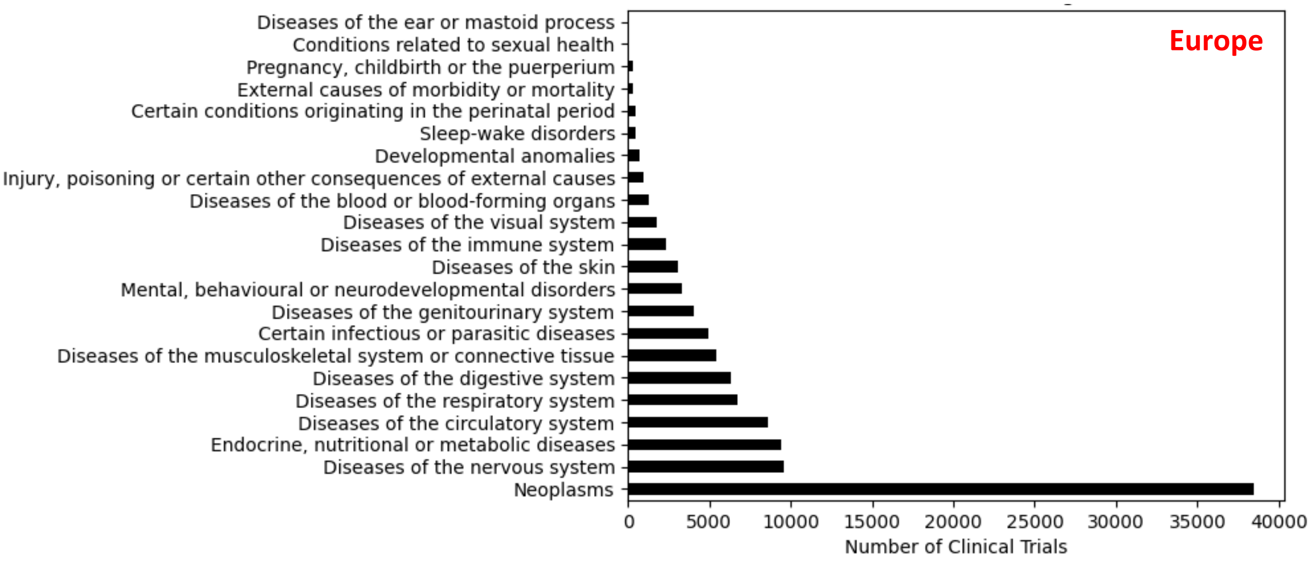

Figure S6. Detailed overview of the health conditions targeted in clinical trials with drug interventions in the US (top) and Europe (bottom). Some clinical trials are counted twice if they covered more than one type of health condition. Counts of clinical trials with health conditions that could not be identified through the current methods are excluded.

Rare Disease. Pharmaceutical innovation in high SDI countries is increasingly focusing on rare diseases. The US Orphan Drug Act defines a rare disease as affecting fewer than 200,000 people total in the United States, or if the cost of developing a drug and making it available in the United States for such diseases or conditions will exceed any potential profits from its sale.[3]

Between 2005 and 2009, 12.6% of US clinical trials were targeted towards rare diseases, which increased to 15.9% in the 2020–2024 period (Fig. S3). In contrast, the global population prevalence of rare diseases is estimated to be between 3.5–5.9%.[23] Values in the literature support the current findings. Firstly, 40% of the drugs approved by the FDA are orphan drugs.[12] In terms of market activity, approximately one fifth of worldwide prescription sales are expected to be captured by orphan drugs in 2024, amounting to $242 billion.[24] Moreover, from 2019 to 2024, orphan drug sales are expected to grow at a compound annual growth rate of 12.3%, nearly double the rate seen for the rest of the drug market.[24]

Three key underlying factors were identified for the observed market emphasis on treating low prevalence diseases. First, rare diseases have additional market exclusivities and monetary subsidies offered by both government and non-government organizations to stimulate research in niche fields which otherwise may not offer much financial incentive for investment. For example, The Orphan Drug Act was established in the US in 1983 to help promote the development of therapeutic interventions for patients with rare disease and has led to over 5000 orphan drug designations,[25] as well as the approval of 491 new orphan drugs between 1990 and 2022.[26] The effect of the Orphan Drug Act in creating incentive for orphan drug development has been remarkable, considering that less than 10 orphan drugs were brought to the market in the decade prior to its inception.[27,28]

The European Medicines Agency has a similar program for medicines granted an orphan designation, which includes ten years of market exclusivity and fee reductions for regulatory activities, complemented by research grants offered by the European Commission and other sources.[29] Ultimately, the example of rare diseases demonstrates the success of government-organized financial and regulatory incentives in driving innovation activity in rare diseases and conditions.

The second factor is that low prevalence health conditions often lack price caps. In their study comparing treatment costs of new orphan and non-orphan drugs approved by the FDA from 2017 to 2021, Althobaiti et al. (2023) found that the median treatment cost for non-orphan drugs was $12,798 USD, compared to $218,872 USD for orphan drugs – approximately 17 times higher.[30]

Indeed, the financial burden on patients and healthcare payers has raised concerns about pricing and affordability of orphan drugs.[31] To mitigate the high cost for vulnerable populations, the 340B program was created by US Congress in 1992, requiring pharmaceutical manufacturers to provide discounts for outpatient prescriptions serving high numbers of uninsured and poor patients.[30] However, the 2010 Affordable Care Act (ACA) excluded orphan drugs from the 340B program.[32] Manufacturers seeking to take advantage of this exception have identified new uses for existing common drugs to obtain orphan drug status from the FDA, as seen with the arthritis-treating blockbuster drug Humira.[33] The lack of price caps coupled with certain guaranteed federal funding such as Medicare coverage drives the high treatment costs for rare diseases and thus provides a strong financial incentive for actors involved in drug development to target rare diseases.

The final factor which gears innovation towards treating niche health conditions is the lack of competition. Whereas other treatment options may exist for highly prevalent health conditions, low prevalence medical problems such as orphan diseases do not have many competing therapeutic options. As such, actors in the drug development pipeline have better opportunities in monopolizing niche therapeutic areas.

Text S6. Supporting information on Meadows' (1999) leverage points for complex systems

In 1999, Meadows proposed a framework outlining twelve types of leverage points found in complex systems, ranging in their degree of effectiveness.[34] The aim of the approach is to better strategize efforts towards achieving desired outcomes within complex systems. The current study adopts a strategic approach based on Meadows' framework, encompassing six types of actionable measures in order of increasing leverage: information flows, financial incentives, rules of the system, structure of the system, goals of the system, and mindset & paradigm. We contend that the lower-level targets are necessary as steppingstones towards leveraging higher level intervention points.

In this section, we analyze each of these leverage points and describe their relevance, drawing on selected examples. Based on this analysis, we then identified and organized roles for individual stakeholders, which are presented in the main text.

Mindset & Paradigm. Changes to mindset & paradigm represents the strongest leverage point of the system, albeit the most abstract. Paradigms describe society's beliefs, assumptions, and values about how the world works, and largely influence how people think, behave, and make decisions. As Meadows (1999) describes, "paradigms are the sources of systems"; from shared social agreements come system goals, structures, rules, financial mechanisms, and information flows.

In the context of pharmaceutical lock-in, the actionable measure for changing mindset & paradigm is the fundamental integration of environmental safety and sustainability with monetary profit as the incentives driving the drug development system. Designing win-win solutions is the goal, where actors can practice environmental safety and sustainability in their businesses without making significant financial sacrifices, or even gain extra profit from making the environmentally safe and sustainable choice.

Goals of the System. Changing the mindset and paradigm of the pharmaceutical world is underpinned by specific changes in goals of the system. The current pharmaceutical industry ecosystem is largely driven by short-term goals, in that the actors involved in the drug development pipeline focus on maximizing financial benefits within a narrow time scope, and on getting safe and effective life-saving medicines to patients as quickly as possible.

The current goals of the system fail to internalize the long-term environmental costs to society associated with producing and using today’s drugs. Going forward, internalizing these negative environmental externalities into the market decision-making processes is essential to effectively greening the pharmacy. Summarized in simple terms, there is a need to change the overall systemic goal from short-term gains towards long-term investment in public health and environmental protection.

Specific to lock-in 1, this can be framed as changing the current goal of making as many safe and effective new drugs as possible towards making as many safe, effective, *and* environmentally sustainable new drugs as possible (one possible strategy, which could be explored in further detail,

could be shifting from traditional APIs towards biologics). Lock-in 2 shares the same current goal as lock-in 1, but in addition to making new drugs, the improvement of existing drugs (specifically with respect to environmental safety and sustainability) should also be part of the overarching goal of the pharmaceutical system.

Structure of the System. Key to addressing lock-in 1 is bridging the gap between actors responsible for early environmental safety and sustainability testing and considerations (i.e., academia and biotech start-ups) and those who have the resources to do so (i.e., big pharma). Linking these two groups of actors earlier in the pipeline (i.e., during drug discovery when major changes to drug structure can still be made) is a significant change to the current operating structure of drug development (i.e., big pharma tends to buy out start-ups shortly before or during the early stages of clinical trials).

With respect to addressing lock-in 2, the current system should change in structure to meet the change in the mindset and paradigm of integrating environmental safety and sustainability with monetary profit. To meet this overarching goal, the new system could financially reward actors who improve the environmental safety and sustainability of existing drugs.

Rules of the System. This category of leverage points refers to legislative and policy frameworks. The patent system is a core motivator for many of the market dynamics seen in the current pharmaceutical world, as the ability to keep market exclusivity and thus profits largely depend on actors' abilities to maintain patent protection. As such, patents present a potential tool in leveraging pharmaceutical market dynamics.

The current pharmaceutical patent system rewards inventions leading to medical breakthroughs and not inventions leading to enhanced environmental safety and sustainability. Again, this connects back to the need for changes in the overarching goals of the system; the existing system is focused on short-term financial gains and medical treatment of patients. Environmental improvements are not rewarded in the current system.

Nevertheless, patents are an important leverage point, and additional market exclusivities could be offered for drugs with certifiable sustainable design. For example, applications for new active pharmaceutical ingredients (APIs) which are certified environmentally safe and sustainable could

benefit from additional patent time.[35] Seeing as even just a year of added market exclusivity can translate to millions of dollars of revenue, especially for blockbuster drugs, this creates a downstream financial incentive for actors to prioritize environmental safety and sustainability. This touches upon lock-in 1, which requires a shift in prioritization of environmental safety and sustainability testing to earlier stages.

For lock-in 2, which is about incentivizing the improvement of existing drugs, patent-related solutions are more nuanced and case-specific. An environmental safety and sustainability-related patent extension could be an attractive choice for drugs nearing their patent expiration. If a drug has already lost its exclusivity and generics have entered the market, the filing of new secondary patents (such as new manufacturing methods) offers little to no benefit. In such cases, a significant, innovative change to the chemical would be needed, which could lead to new market authorization and exclusivity.

Fast-tracking of regulatory approval is another potential tool with which sustainable drug design can be incentivized.[35] Accelerated approvals already exist in the FDA for promising therapies which offer benefits over existing options for treating life-threatening or serious conditions through the *Breakthrough Therapy* designation and *Fast-Track* process. While environmental safety and sustainability of a drug may not warrant the urgency of such fast-track processes, priority review is a potentially suitable designation which could incentivize the development of greener drugs. Compared to 10 months under standard review, a drug designated under priority review receives feedback from the FDA within 6 months.[36] Some green patent application fast-tracking systems already exist, but it is unclear if they apply to pharmaceuticals.[37]

Mandated environmental risk assessments (ERA) as part of drug approval applications is another potential tool for prioritizing environmental safety and sustainability considerations in the drug development pipeline. Some regulatory bodies have already implemented assessments of the environmental impact of pharmaceuticals in their approval processes, such as the European Medicines Agency and the FDA. In the EU, environmental risk currently has no relevance to the approval of the pharmaceutical, and there are no penalties for non-compliance for completion of the ERA.[38]

In April 2023, the European Commission adopted a proposal for a new Directive and a new Regulation, which revise and replace the existing general pharmaceutical legislation.[39,40] Under this proposal, authorities could reject marketing authorization applications if the accompanying ERA is inadequate or if environmental risks are insufficiently addressed. Authorities could also impose conditions on approved medicines, such as restricting them to prescription-only use or requiring additional post-authorization ERAs. If a pharmaceutical poses a serious environmental risk, authorities would be able to suspend, revoke, or modify marketing authorizations.

Finally, a major contributing factor to lock-in 1 is that current pharmaceutical regulations and approval processes focus on the later stages of drug development, i.e., clinical trials. Mandating earlier environmental safety and sustainability testing and considerations as a pre-condition to enter clinical trials could be a helpful strategy towards re-aligning environmental safety and sustainability prioritization in drug development, thus helping address lock-in 1.

Financial Incentives. Financial incentives are a powerful tool that can be used to drive systematic change in the pharmaceutical world. Currently, negative environmental externalities associated with the pharmaceutical industry are being absorbed by the public. What is needed for meaningful change is for the internalization of negative externalities by the industry itself (i.e., polluter-pays-principle). This approach is two-fold, starting with start-ups and academia.

Early players, such as start-ups and academics, get a large part of their funding from public sources and grants. Especially for researchers in academia, government subsidies can motivate certain kinds of research through direct monetary compensation. For instance, subsidies could be granted to actors who invest research efforts in the improvement of environmental safety and sustainability of existing drugs (i.e., addressing lock-in 2), or to those investing in new green drugs (i.e., addressing lock-in 1). Financial institutions also present an opportunity where environmental safety and sustainability could be an additional criterion for investment choice.

For big pharma companies, a key leverage point lies within shareholder behavior. The financial security of publicly traded pharmaceutical companies depends largely on shareholders. However, rankings and recommendations by investment banking groups are critical drivers of shareholder activity. Environmental liability could be embedded within these rankings and recommendations. If shareholders preferentially invest in companies with environmentally sustainable values and

actions, this creates a further demand for eco-friendly behavior from big pharma and ultimately leads to a positive feedback loop.

It is important to note that a clear definition of an “environmentally safe and sustainable” drug would be essential to promote fair play (i.e., to avoid “greenwashing”). A certification system for environmental safety and sustainability of new pharmaceuticals, perhaps using a scoring system, could be established for this purpose and could be utilized by both subsidy grantors and investors. Such a system could be aligned with the broader Safe and Sustainable by Design (SSbD) framework proposed by the European Commission, which aims to guide innovation towards inherently safer and more sustainable chemicals and materials from the outset. Recent work by Puhlmann et al. (2024) suggested that the SSbD framework could be conceptually extended to the pharmaceutical industry.[41]

Information Flows. Changes to access and availability to information is the sixth and final class of actionable measures. Educational campaigns for the general public can increase awareness of the direct and indirect costs of pharmaceutical pollution and the importance of addressing this issue. Consumers may have a choice in the types of pharmaceuticals they consume (e.g., selectivity towards environmental safety and sustainability) and how to properly dispose of them.

Guidance and training for medical practitioners and pharmacists could lead to more sustainable prescription practices. This could entail, for example, only prescribing drugs when suitable non-pharmacotherapy alternative treatments have been exhausted, or selectively prescribing more eco-friendly pharmaceuticals when possible.[42]

Changes in information flows can be a useful tool for meeting higher level actionable measures – specifically, bridging the gap between actors responsible for early environmental testing (i.e., academia/start-ups) and those who have the resources to do so (i.e., big pharma), which was proposed as a structural change needed to address lock-in 1. Potential changes to information flows to help bridge this gap include enabling open access to big pharma data and tools, as well as the provision of resources and training from big pharma to early actors for environmental safety and sustainability testing.[35] Moreover, training within academic institutions in green chemistry principles could become a prerequisite for new graduate students and faculty.

SI References

(1) NIH. *Why Should I Register and Submit Results?*. U.S. National Library of Medicine Clinicaltrials.gov. https://classic.clinicaltrials.gov/ct2/manage-recs/background#RegLawPolicies (accessed 2024-04-06).

(2) Top Publicly Traded Pharmaceutical Companies by Revenue. https://companiesmarketcap.com/pharmaceuticals/largest-pharmaceutical-companies-by-revenue/ (accessed 2024-12-01).

(3) *Orphan Drug Act*; 1983; pp 2049–2066.

(4) DiMasi, J. A. New Drug Innovation and Pharmaceutical Industry Structure: Trends in the Output of Pharmaceutical Firms. *Ther Innov Regul Sci* **2000**, *34* (4), 1169–1194. https://doi.org/10.1177/009286150003400425.

(5) Jung, Y. L.; Hwang, J.; Yoo, H. S. Disease Burden Metrics and the Innovations of Leading Pharmaceutical Companies: A Global and Regional Comparative Study. *Globalization and Health* **2020**, *16* (1), 80. https://doi.org/10.1186/s12992-020-00610-2.

(6) Liu, A. *Get ready for M&A: Large biopharma companies will have $1.7T in dealmaking firepower next year, analyst says*. Fierce Pharma. https://www.fiercepharma.com/pharma/m-a-large-biotech-pharma-companies-will-have-1-7t-firepower-for-dealmaking-2022 (accessed 2024-04-06).

(7) Bushak, L. *Pharma M&A rebounded in '23, set to grow in '24, report finds*. MM+M - Medical Marketing and Media. https://www.mmm-online.com/home/channel/pharma-merger-acquisition-rebounded-set-to-grow/ (accessed 2024-04-06).

(8) Wajid, A.; Khan, K. H.; Handa, H. Innovations Through Mergers and Acquisitions in the Pharmaceutical Sector. In *Technological Innovations for Sustainability and Business Growth*; IGI Global, 2020; pp 91–104.

(9) Koenig, M.; Mezick, E. Impact of Mergers & Acquisitions on Research Productivity within the Pharmaceutical Industry. *Scientometrics* **2004**, *59*, 157–169. https://doi.org/10.1023/B:SCIE.0000013304.40957.0d.

(10) *Data & Market Exclusivity As Incentives in Drug Development*. Scendea. https://www.scendea.com/articles/blog-post-title-one-25srn-58l3m-hef63 (accessed 2024-04-06).

(11) Research, C. for D. E. and. Generic Drug Facts. *FDA* **2021**.

(12) Feldman, R. May Your Drug Price Be Evergreen. *Journal of Law and the Biosciences* **2018**, *5* (3), 590–647. https://doi.org/10.1093/jlb/lsy022.
(13) Berndt, E. R.; Aitken, M. L. *Brand Loyalty, Generic Entry and Price Competition in Pharmaceuticals in the Quarter Century After the 1984 Waxman-Hatch Legislation*; NBER working paper series; National Bureau of Economic Research: Cambridge, Mass, 2010.
(14) Higgins, M. J.; Kronlund, M. J.; Park, J. M.; Pollet, J. *The Role of Assets In Place: Loss of Market Exclusivity and Investment*; NBER working paper series; National Bureau of Economic Research: Cambridge, Mass, 2020.
(15) Cockburn, I. M.; MacGarvie, M. J.; Muller, E. *Patent Thickets, Licensing and Innovative Performance*; ZEW Discussion Papers; 08–101; Zentrum für Europäische Wirtschaftsforschung (ZEW): Mannheim, 2008. https://ftp.zew.de/pub/zew-docs/dp/dp08101.pdf.
(16) Cooper, R. *How Big Pharma Rigged the Patent System*. The American Prospect. https://prospect.org/api/content/30a3e5d8-03c0-11ee-aa27-12163087a831/ (accessed 2024-04-06).
(17) Gupta, H.; Kumar, S.; Roy, S. K.; Gaud, R. S. Patent Protection Strategies. *J Pharm Bioallied Sci* **2010**, *2* (1), 2–7. https://doi.org/10.4103/0975-7406.62694.
(18) KPMG. Profit Margin for Generics Manufacturers Worldwide from FY 2016 to FY 2019, 2020. https://www.statista.com/statistics/1248196/profit-margin-for-generics-manufacturers-worldwide/.
(19) Sullivan, R. The Gross Imbalances of Cancer Research Must Be Addressed. *Nature* **2023**, *621* (7977), S15–S15. https://doi.org/10.1038/d41586-023-02609-2.
(20) Cancer Is a Curse, but Also a Growth Market for Investors. *The Economist*. https://www.economist.com/finance-and-economics/2018/02/01/cancer-is-a-curse-but-also-a-growth-market-for-investors?utm_medium=cpc.adword.pd&utm_source=google&ppccampaignID=18151738051&ppcadID=&utm_campaign=a.22brand_pmax&utm_content=conversion.direct-response.anonymous&gad_source=1&gclid=CjwKCAjwte-vBhBFEiwAQSv_xedVZAUh2VFpBzPmDGnpVuUeGiTTM--30-248y0NgufNYmMBKnu-KxoCResQAvD_BwE&gclsrc=aw.ds (accessed 2024-04-07).

(21) Pfizer Inc. *Pfizer Invests $43 Billion to Battle Cancer*. Pfizer. https://www.pfizer.com/news/press-release/press-release-detail/pfizer-invests-43-billion-battle-cancer (accessed 2024-04-07).
(22) González Peña, O. I.; López Zavala, M. Á.; Cabral Ruelas, H. Pharmaceuticals Market, Consumption Trends and Disease Incidence Are Not Driving the Pharmaceutical Research on Water and Wastewater. *Int J Environ Res Public Health* **2021**, *18* (5), 2532. https://doi.org/10.3390/ijerph18052532.
(23) Nguengang Wakap, S.; Lambert, D. M.; Olry, A.; Rodwell, C.; Gueydan, C.; Lanneau, V.; Murphy, D.; Le Cam, Y.; Rath, A. Estimating Cumulative Point Prevalence of Rare Diseases: Analysis of the Orphanet Database. *Eur J Hum Genet* **2020**, *28* (2), 165–173. https://doi.org/10.1038/s41431-019-0508-0.
(24) Villa, F.; Di Filippo, A.; Pierantozzi, A.; Genazzani, A.; Addis, A.; Trifirò, G.; Cangini, A.; Tafuri, G.; Settesoldi, D.; Trotta, F. Orphan Drug Prices and Epidemiology of Rare Diseases: A Cross-Sectional Study in Italy in the Years 2014–2019. *Front. Med.* **2022**, *9*. https://doi.org/10.3389/fmed.2022.820757.
(25) Miller, K. L.; Kraft, S.; Ipe, A.; Fermaglich, L. Drugs and Biologics Receiving FDA Orphan Drug Designation: An Analysis of the Most Frequently Designated Products and Their Repositioning Strategies. *Expert Opin Orphan Drugs* **2022**, *9* (11–12), 265–272. https://doi.org/10.1080/21678707.2021.2047021.
(26) Miller, K. L.; Lanthier, M. Orphan Drug Label Expansions: Analysis Of Subsequent Rare And Common Indication Approvals. *Health Affairs* **2024**, *43* (1), 18–26. https://doi.org/10.1377/hlthaff.2023.00219.
(27) Medicine, T. L. R. Victory for a Rare Alliance. *The Lancet Respiratory Medicine* **2013**, *1* (6), 423. https://doi.org/10.1016/S2213-2600(13)70167-X.
(28) Seoane-Vazquez, E.; Rodriguez-Monguio, R.; Szeinbach, S. L.; Visaria, J. Incentives for Orphan Drug Research and Development in the United States. *Orphanet J Rare Dis* **2008**, *3*, 33. https://doi.org/10.1186/1750-1172-3-33.
(29) European Medicines Agency. *Orphan incentives*. European Medicines Agency. https://www.ema.europa.eu/en/human-regulatory-overview/research-and-development/orphan-designation-research-and-development/orphan-incentives (accessed 2024-04-07).

(30) Althobaiti, H.; Seoane-Vazquez, E.; Brown, L. M.; Fleming, M. L.; Rodriguez-Monguio, R. Disentangling the Cost of Orphan Drugs Marketed in the United States. *Healthcare (Basel)* **2023**, *11* (4), 558. https://doi.org/10.3390/healthcare11040558.
(31) Côté, A.; Keating, B. What Is Wrong with Orphan Drug Policies? *Value Health* **2012**, *15* (8), 1185–1191. https://doi.org/10.1016/j.jval.2012.09.004.
(32) Yang, Y. T.; Chen, B.; Bennett, C. L. Federal 340B Program Payment Scheme for Drugs Designated As Orphan Products: Congressional Clarification Needed to Close the Government-Industry Revolving Door. *J Clin Oncol* **2016**, *34* (36), 4320–4322. https://doi.org/10.1200/JCO.2016.68.2989.
(33) Tribble, S. J.; Lupkin, S. *Drugmakers Manipulate Orphan Drug Rules To Create Prized Monopolies*. KFF Health News. https://kffhealthnews.org/news/drugmakers-manipulate-orphan-drug-rules-to-create-prized-monopolies/ (accessed 2024-04-07).
(34) Meadows, D. *Leverage Points: Places to Intervene in a System*; The Sustainability Institute, 1999. https://1a0c26.p3cdn2.secureserver.net/wp-content/userfiles/Leverage_Points.pdf (accessed 2024-04-06).
(35) Moermond, C. T. A.; Puhlmann, N.; Brown, A. R.; Owen, S. F.; Ryan, J.; Snape, J.; Venhuis, B. J.; Kümmerer, K. GREENER Pharmaceuticals for More Sustainable Healthcare. *Environ. Sci. Technol. Lett.* **2022**, *9* (9), 699–705. https://doi.org/10.1021/acs.estlett.2c00446.
(36) FDA. *Priority Review*. Priority Review. https://www.fda.gov/patients/fast-track-breakthrough-therapy-accelerated-approval-priority-review/priority-review (accessed 2024-04-09).
(37) Dechezleprêtre, A. *Fast-Tracking Green Patent Applications: An Empirical Analysis*; ; ICTSD Programme on Innovation, Technology and Intellectual Property; Issue Paper No. 37; International Centre for Trade and Sustainable Development: Geneva, 2013.
(38) Fumagalli, D. Environmental Risk and Market Approval for Human Pharmaceuticals. *Monash Bioeth Rev* **2024**, *42* (Suppl 1), 105–124. https://doi.org/10.1007/s40592-024-00195-1.
(39) European Commission, Directorate-General for Health and Food Safety. *Proposal for a REGULATION OF THE EUROPEAN PARLIAMENT AND OF THE COUNCIL Laying down Union Procedures for the Authorisation and Supervision of Medicinal Products for*

Human Use and Establishing Rules Governing the European Medicines Agency, Amending Regulation (EC) No 1394/2007 and Regulation (EU) No 536/2014 and Repealing Regulation (EC) No 726/2004, Regulation (EC) No 141/2000 and Regulation (EC) No 1901/2006; 2023. https://eur-lex.europa.eu/legal-content/EN/TXT/?uri=CELEX%3A52023PC0193 (accessed 2025-07-17).

(40) European Commission, Directorate-General for Health and Food Safety. *Proposal for a DIRECTIVE OF THE EUROPEAN PARLIAMENT AND OF THE COUNCIL on the Union Code Relating to Medicinal Products for Human Use, and Repealing Directive 2001/83/EC and Directive 2009/35/EC*; 2023. https://eur-lex.europa.eu/legal-content/EN/TXT/?uri=CELEX%3A52023PC0192 (accessed 2025-07-17).

(41) Puhlmann, N.; Abbate, E.; Kümmerer, K.; Oomen, A. G.; Ragas, A. M. J.; Moermond, C. Applicability of the European Commission's Framework on *Safe and Sustainable by Design* to the Pharmaceutical Sector. *Sustainable Chemistry and Pharmacy* **2024**, *42*, 101845. https://doi.org/10.1016/j.scp.2024.101845.

(42) Orive, G.; Lertxundi, U.; Brodin, T.; Manning, P. Greening the Pharmacy. *Science* **2022**, *377* (6603), 259–260. https://doi.org/10.1126/science.abp9554.
